# miR-19 Is a Potential Clinical Biomarker for Gastrointestinal Malignancy: A Systematic Review and Meta-analysis

**DOI:** 10.1155/2020/2810150

**Published:** 2020-09-10

**Authors:** Xiaoxu Song, Wenyi Li, Peng Shen, Big Bai, Lin-Lin Cao

**Affiliations:** ^1^Department of Clinical Laboratory, Wu'an First People's Hospital, Handan, China; ^2^Department of Clinical Laboratory, Peking University People's Hospital, Beijing, China

## Abstract

**Objectives:**

To assess the expression and clinical value of miR-19 in gastrointestinal malignancy. *Setting*. Embase, Web of Science, PubMed, and other databases were retrieved to screen out relevant studies until December 31, 2019. *Participants*. Gastrointestinal cancer patients with the description of miR-19 expression, as well as the correlation between miR-19 and clinicopathological characteristics or prognosis. *Main Outcome Measures*. Pooled odds ratio (OR) or hazard ratio (HR) with 95% confidence interval (CI) was obtained to determine miR-19 expression in gastrointestinal malignancy and the association between miR-19 and patients' clinical characteristics and survival.

**Results:**

Thirty-seven studies were included in this study. miR-19 levels in gastrointestinal malignancy, especially in hepatocellular (OR = 4.88, 95%  CI = 2.38‐9.99), colorectal (OR = 4.81, 95%  CI = 2.38‐9.72), and pancreatic (OR = 5.12, 95%  CI = 2.43‐10.78) cancers, were significantly overexpressed, and miR-19 was tightly related to some clinicopathological characteristics, such as lymph node metastasis (OR = 1.74, 95%  CI = 1.05‐2.86). Although gastrointestinal cancer patients with low and high miR-19 expression had comparable OS (overall survival) and DFS (disease-free survival), subgroup analyses showed that patients with high miR-19 presented better DFS than those with low miR-19 in liver cancer (HR = 0.46, 95%  CI = 0.30‐0.71).

**Conclusions:**

miR-19 might be a potential progression and prognostic biomarker for gastrointestinal malignancy.

## 1. Background

Gastrointestinal malignancy is extremely harmful to humans, including gastric, pancreatic, esophageal, liver, and colorectal cancers and other types of cancer in the digestive tract. Their morbidity and mortality rates are really high, especially in less-developed countries [1]. Although great progress has been achieved in early diagnosis and therapy during the past few decades, the overall survival (OS) for gastrointestinal malignancy is still unsatisfactory [2]. Therefore, it is essential to identify novel biomarkers for patients' early diagnosis and better prognosis.

MicroRNAs (miRNAs) are a kind of small noncoding RNA, which can modulate gene expression by cleaving targeted messenger RNA (mRNA) or repressing translation [3]. A number of studies have reported that miRNAs show the potential to be novel cancer biomarkers for early detection of cancer [4–6]. MicroRNA-19 (miR-19), which is one member of the large miRNA family, has been demonstrated to be tightly correlated with gastrointestinal malignancy [7–10]. However, the exact role of miR-19 in gastrointestinal malignancy is still unclear.

In the present study, a systematic review and meta-analysis was carried out to assess the association of miR-19 with gastrointestinal cancers. At first, miR-19 expression in gastrointestinal cancer tissue and normal tissue was compared, and then, the correlation of the miR-19 level with several clinical characteristics was evaluated. In addition, the role of miR-19 in prognosis for patients with gastrointestinal cancers was also determined.

## 2. Methods

### 2.1. Search Strategy and Inclusion Criteria

Original researches reporting the association of miR-19 with the progression or prognosis of gastrointestinal cancers were retrieved in Embase, Web of Science, PubMed, and other databases until December 31, 2019. No language restriction was used. We selected studies according to the following keywords: “miR-19”, “microRNA-19”, or “miRNA-19” for miR-19; “colorectal carcinoma” or “colorectal cancer” for colorectal cancer; “esophageal cancer” or “esophagus neoplasm” for esophageal cancer; “gastric neoplasm”, “gastric cancer”, or “stomach cancer” for gastric cancer; “liver cancer”, “hepatocellular carcinoma”, or “hepatocellular cancer” for liver cancer; and “pancreatic neoplasm” or “pancreatic cancer” for pancreatic cancer.

Then, full texts of the relevant studies were evaluated deeply. The inclusion criteria were the following: (1) the expression level of miR-19 was detected by PCR, (2) the clinicopathological parameters or patient survival of gastrointestinal cancers were investigated, and (3) the association of miR-19 with clinicopathological parameters or patient survival was assessed. Studies were excluded if (1) they were not original articles, such as letters, case reports, or reviews; (2) they were focusing on cancer cells or animal models, rather than human samples; or (3) the full texts were not available. Two authors, Xiaoxu Song and Lin-Lin Cao, performed the evaluations independently, and disagreement was settled according to the original article.

### 2.2. Data Extraction

Data were extracted by Xiaoxu Song and Wenyi Li independently. The extracted information included the first author's name, country, publication year, age and number of patients, the method of miR-19 detection, cut-off point, histology, clinical stage, and survival. If the cut-off point of miR-19 was not described in the studies, the mean value was used as the cut-off point. If there was only a histogram and no original data for miR-19 expression were provided, Engauge Digitizer 4.1 was applied to extract the needed data. In addition, Engauge Digitizer 4.1 was also used for the survival data if there were only Kaplan-Meier curves in the included studies [11].

### 2.3. Quality Assessment

The quality evaluation of the retrieved studies was completed by Xiaoxu Song and Wenyi Li independently based on the Newcastle-Ottawa Scale (NOS), which includes three parts: sample selection, comparability, and exposure ascertainment.

### 2.4. Statistical Analysis

All analyses were carried out with Review Manager 5.3 (Cochrane Collaboration, Oxford, UK). The odds ratio (OR) with 95% confidence interval (CI) was calculated to compare miR-19 levels between the tumor group and the control group and to analyze the correlation between miR-19 and clinicopathologic characters of gastrointestinal cancers. The association of miR-19 levels with patient prognosis was determined using the hazard ratio (HR) with 95% confidence interval (CI). The model of random effect was used if *I*^2^ > 50%; otherwise, the model of fixed effect was applied (*I*^2^ ≤ 50%). *P* < 0.05 was statistically significant. The funnel plot was depicted to determine publication bias.

### 2.5. Patient and Public Involvement

There is no patient involved.

## 3. Results

### 3.1. Description of the Included Cohorts

**Figure 1:**
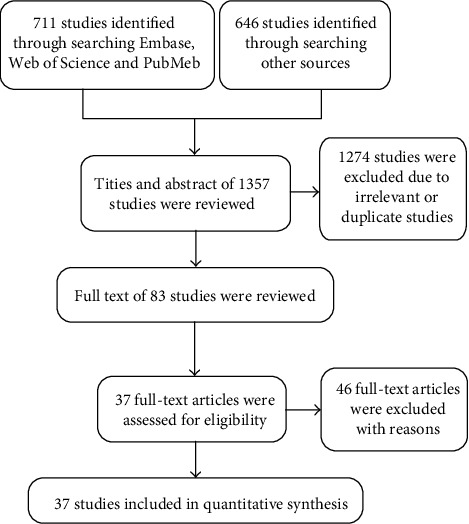
Methodological flow chart of study selection.

In this analysis, 711 studies were identified through searching Embase, PubMed, and Web of Science, and 646 studies were identified in other databases. In total, 1357 studies were found initially (Figure 1). Then, 1274 studies were excluded due to their irrelevance or duplication after checking their titles and abstracts. The remaining 83 studies were read carefully in full text, and 46 were excluded as well because of the following two reasons: (1) there was no data of human samples but only cell lines or animal models or (2) relevant data was not available. Finally, 37 studies [7, 9, 10, 12–45] were included in this analysis, including 12 studies focusing on colorectal cancer, 11 studies focusing on gastric cancer, 8 studies focusing on liver cancer, 2 study focusing on esophageal cancer, and 4 studies focusing on pancreatic cancer. Some characteristics and results of these studies are described in Table 1. Totally, 3472 cases were included in this analysis. All these studies used real-time polymerase chain reaction (RT-PCR) for miR-19 detection. NOS evaluation results suggested high quality of all the studies (Table 2).

### 3.2. miR-19 Levels in Gastrointestinal Cancers Were Higher than Those in Noncancerous Controls

**Figure 2:**
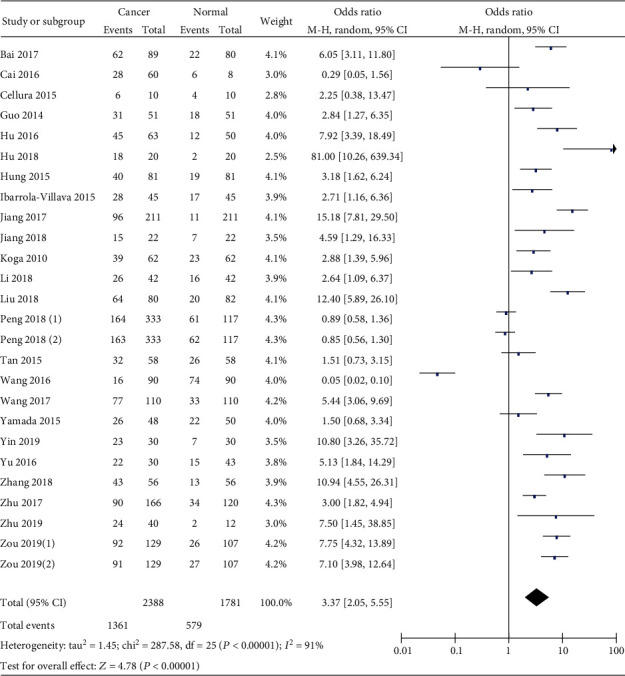
Forest plot of odds ratio (OR). Relative miR-19 abundance of overall gastrointestinal malignancy in comparison to noncancerous controls.

Most of the included studies have compared miR-19 levels between gastrointestinal cancers and noncancerous controls, including 7 studies focusing on colorectal cancer, 8 studies focusing on gastric cancer, 5 studies focusing on liver cancer, 3 studies focusing on pancreatic cancer, and only 1 study focusing on esophageal cancer. The result is shown in Figure 2 (OR = 3.37, 95%  CI = 2.05‐5.55), suggesting that miR-19 levels in gastrointestinal malignancy were higher than those in controls.

**Figure 3:**
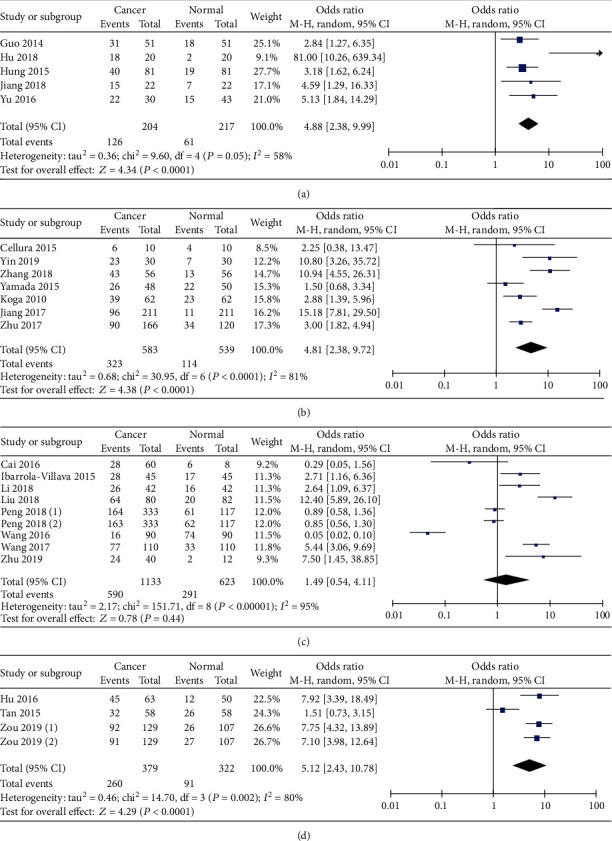
Forest plot of odds ratio (OR). (a) Comparison of the expression level of miR-19 between liver cancer and control. (b) Comparison of the expression level of miR-19 between colorectal cancer and control. (c) Comparison of the expression level of miR-19 between gastric cancer and control. (d) Comparison of the expression level of miR-19 between pancreatic cancer and control.

Then, we carried out subgroup analysis according to different cancers. As shown in Figure 3(a), miR-19 levels in liver cancer were higher than those in the control group (OR = 4.88, 95%  CI = 2.38‐9.99). Similar results were found in colorectal cancer (OR = 4.81, 95%  CI = 2.38‐9.72) and pancreatic cancer (OR = 5.12, 95% CI = 2.43‐10.78) (Figures 3(b) and 3(d)). However, no significant distinction existed between gastric cancer and noncancerous group (Figure 3(c)). There was only one study focusing on esophageal cancer. Taken together, these data indicate that miR-19 levels in gastrointestinal cancers, especially colorectal, liver, and pancreatic cancers, were higher than those in noncancerous controls.

### 3.3. Association of miR-19 Expression with the Clinical Characteristics of Patients with Gastrointestinal Malignancy

**Figure 4:**
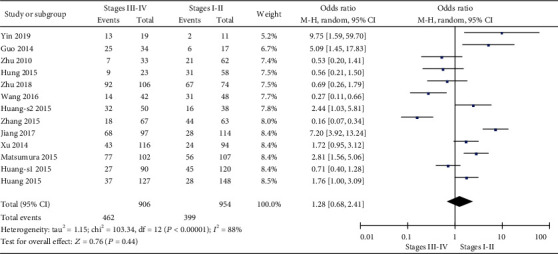
Forest plot of odds ratio (OR). Association between miR-19 expression and tumor stage in overall gastrointestinal malignancy.

**Figure 5:**
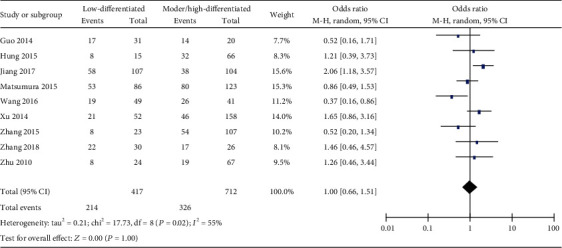
Forest plot of odds ratio (OR). Association between miR-19 expression and tumor differentiation degree in overall gastrointestinal malignancy.

**Figure 6:**
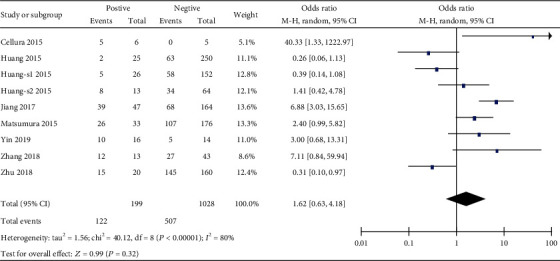
Forest plot of odds ratio (OR). Association between miR-19 expression and distant metastasis in overall gastrointestinal malignancy.

**Figure 7:**
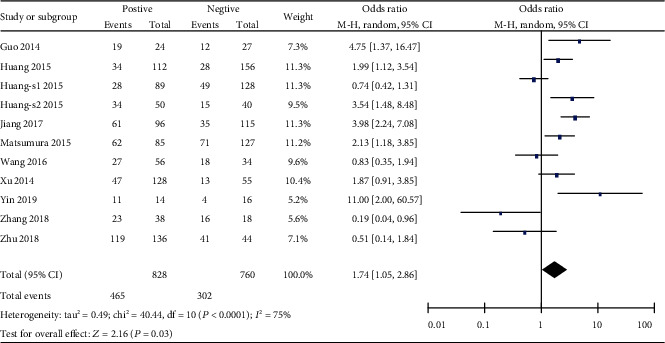
Forest plot of odds ratio (OR). Association between miR-19 expression and lymph node metastasis in overall gastrointestinal malignancy.

Next, we determined the correlation between miR-19 and the clinicopathologic characteristics of patients with gastrointestinal malignancy. Unfortunately, there is no significant correlation between the miR-19 level and some clinical features, such as the tumor stage, differentiation degree, or distant metastasis of overall gastrointestinal cancers (Figures 4–6). Interestingly, we discovered that miR-19 levels were upregulated in lymph node metastasis-positive patients (OR = 1.74, 95%  CI = 1.05‐2.86) (Figure 7).

The results of subgroup analyses are displayed in Table 3. miR-19 levels in stages III-IV were higher than those in stage I-II colorectal cancer (OR = 2.74, 95%  CI = 1.45‐5.18). In addition, the miR-19 expression levels were lower in low-differentiated gastric tissues than those high-/moderate-differentiated ones (OR = 0.31, 95%  CI = 0.14‐0.70). There is no significant distinction in other analyses, and some analyses were short of studies (0 or 1 study), especially for esophagus and pancreatic cancers. Collectively, there are some relationship between miR-19 levels and clinicopathologic characteristics in gastrointestinal malignancy.

### 3.4. Influence of miR-19 on Clinical Outcome of Gastrointestinal Malignancy

**Figure 8:**
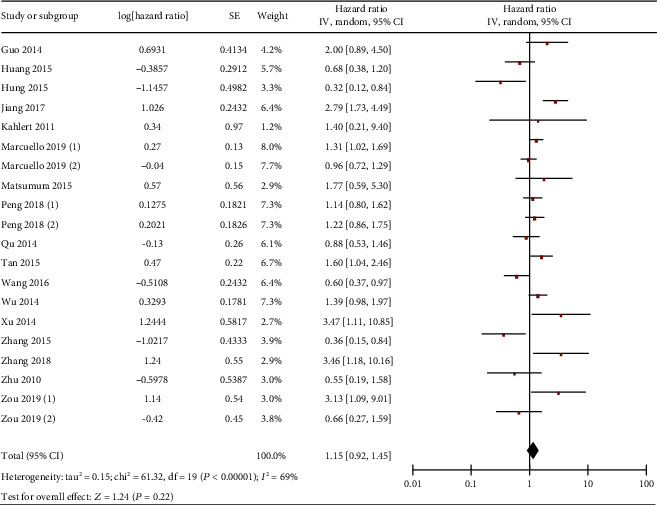
Forest plot of hazard ratio (HR). Association between miR-19 expression and the OS of overall gastrointestinal cancer patients.

**Figure 9:**
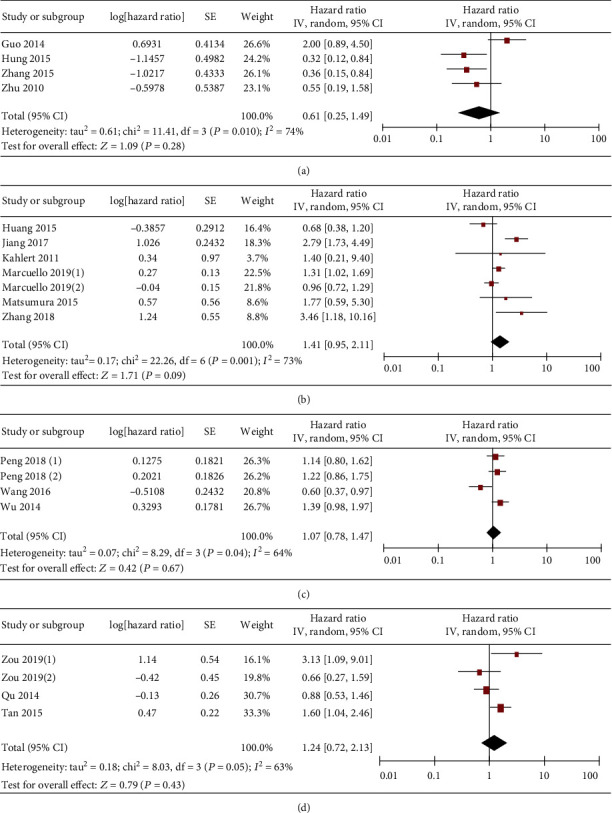
Forest plot of hazard ratio (HR). Association between miR-19 expression and the OS of liver cancer (a), colorectal cancer (b), gastric cancer (c), and pancreatic cancer (d) patients.

Finally, the correlation between miR-19 and OS as well as disease-free survival (DFS) of gastrointestinal malignancy was investigated. Firstly, the analysis result showed that gastrointestinal cancer patients with low and high miR-19 expression showed comparable OS (Figure 8). Similar results were found in subgroup analyses for liver (Figure 9(a)), colorectal (Figure 9(b)), gastric (Figure 9(c)), and pancreatic (Figure 9(d)) cancers.

**Figure 10:**
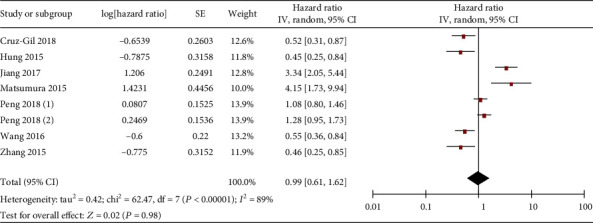
Forest plot of hazard ratio (HR). Association between miR-19 expression and the DFS of overall gastrointestinal cancer patients.

**Figure 11:**
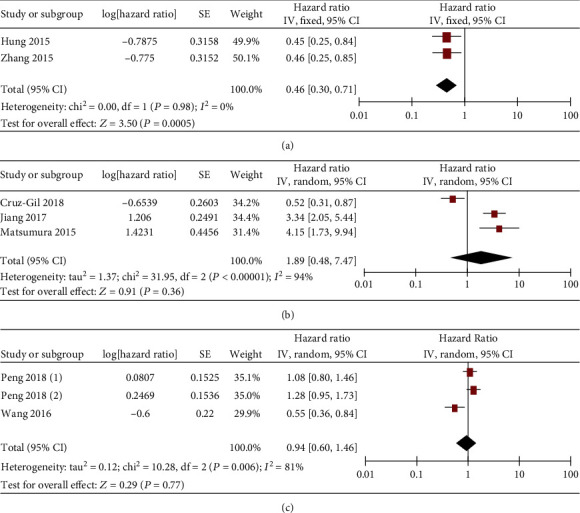
Forest plot of hazard ratio (HR). Association between miR-19 expression and the DFS of liver cancer (a) and colorectal cancer (b).

In addition, gastrointestinal cancer patients with low and high miR-19 expression showed comparable DFS as well (Figure 10). Subgroup analyses showed that the miR-19 level was positively associated with the DFS of liver cancer patients (HR = 0.46, 95%  CI = 0.30‐0.71) (Figure 11(a)), but not colorectal and gastric cancer patients (Figures 11(b) and 11(c)). There was short of study analyzing the DFS of esophageal and pancreatic cancer patients (0 or 1 study).

### 3.5. Sensitivity and Bias Analysis

We conducted sensitivity analysis by removing a cohort one time. Results of meta-analyses were not altered greatly, suggesting the stability of these analyses. In addition, no significant publication biases existed according to the symmetric funnel plots (Supplement Figures. 1-7).

## 4. Discussions

In this study, an analysis of 37 studies revealed a potential role of miR-19 in the progression and prognosis of gastrointestinal cancers. At first, miR-19 levels in gastrointestinal cancers are significantly higher than those in controls. In addition, the association of miR-19 expression with clinical characteristics, such as the clinical stage, tumor differentiation degree, and lymph node and distant metastasis state, was described in subgroup analysis. At last, we depicted that liver cancer patients with higher miR-19 levels showed better DFS than those with low miR-19.

miR-19 expression levels in different gastrointestinal malignancies are inconsistent. For liver and colorectal cancers, most studies showed that miR-19 is overexpressed in cancer patients compared with normal controls. However, miR-19 expression in gastric and pancreatic cancers is controversy. For example, it has been illustrated that the miR-19 levels were upregulated significantly in gastric cancer patients [19, 32], but another study [9] discovered that miR-19 levels were decreased in gastric tumors. In addition, the miR-19 level has been demonstrated to be upregulated in pancreatic cancer [18], but no difference between pancreatic cancer and control was observed in another study [31]. In this study, miR-19 levels in gastrointestinal malignancy were higher than those in the control generally. However, it is necessary to do much more work for pancreatic and esophagus cancers due to the limited number of included studies.

In the present study, significant correlation between miR-19 levels and lymph node metastasis was observed in gastrointestinal malignancy, suggesting the role of miR-19 as a potential biomarker to diagnose patients with lymph node metastasis. Although the correlations between miR-19 and clinical stage, tumor differentiation degree, or distant metastasis state in the overall gastrointestinal malignancy were not significant, subgroup analysis has shown that miR-19 has diagnostic value in specific cancer types. In addition, no correlation between miR-19 and OS or DFS of overall gastrointestinal malignancy was observed, but the miR-19 level was positively correlated with the DFS of liver cancer patients as depicted in subgroup analyses, indicating that miR-19 shows its potential as a prognostic biomarker for liver cancer and would be beneficial for screening out high-risk liver cancer patients.

## 5. Conclusions

This study revealed the clinical significance of the miR-19 level in gastrointestinal malignancy. miR-19 could be a potential clinical biomarker for the progress and survival evaluation for gastrointestinal cancers and used as a new target for gastrointestinal cancer treatment.

## Figures and Tables

**Table 1:** Main characteristics and results of the included studies.

Study	Year	Country or area	Sample number	Age	Detection method	Cut-off point	Histology	Stage	Follow-up period (month)	Survival
Yamada	2015	USA	48	NR	RT-PCR	>median	CRC	NR	NR	NR
Kahlert	2011	Germany	29	NR	RT-PCR	NR	CRC	NR	60	OS, RFS
Cellura	2015	UK	10	NR	RT-PCR	≥median	CRC	NR	NR	NR
Huang	2015	China	275	60 (mean)	RT-PCR	≥0.22	CRC	I-IV	NR	OS
Jiang	2017	China	211	65 (mean)	RT-PCR	>median	CRC	I-IV	59 (median)	OS, DFS
Mastumura	2015	Japan	209	65 (mean)	RT-PCR	NR	CRC	I-IV	60	OS, DFS
Cruz-Gil	2018	Spain	126	NR	RT-PCR	NR	CRC	II-III	NR	DFS
Koga	2010	Japan	62	60 (median)	RT-PCR	>median	CRC	NR	NR	NR
Zhu	2017	China	166	60 (mean)	RT-PCR	>median	CRC	I-IV	NR	NR
Zhang	2018	China	56	60 (mean)	RT-PCR	>median	CRC	I-IV	80	OS
Yin	2019	China	30	50 (mean)	RT-PCR	>median	CRC	I-IV	NR	NR
Marcuello	2019	Spain	59	62 (mean)	RT-PCR	NR	CRC	I-IV	NR	NR
Guo	2014	China	51	50 (mean)	RT-PCR	>median	HCC	I-IV	60	OS
Han	2012	China	105	56.5 (mean)	RT-PCR	NR	HCC	I-IV	80	OS, DFS
Hu	2018	China	20	NR	RT-PCR	>median	HCC	NR	NR	NR
Hung	2015	Taiwan	81	60 (mean)	RT-PCR	≥median	HCC	II-IV	37 (mean)	OS, DFS
Yu	2016	China	43	NR	RT-PCR	≥median	HCC	NR	NR	NR
Zhang	2015	China	130	50 (mean)	RT-PCR	≥median	HCC	I-IV	60	OS, DFS
Zhu	2010	China	95	50 (mean)	RT-PCR	Relative expression > 1.04	HCC	I-III	62.6 (mean)	OS
Jiang	2018	China	22	NR	RT-PCR	≥median	HCC	NR	NR	NR
Cai	2016	China	60	NR	RT-PCR	>median	GC	NR	NR	NR
Li	2014	China	30	50 (mean)	RT-PCR	NR	GC	I-IV	NR	NR
Ibarrola-Villava	2015	Spain	45	NR	RT-PCR	≥median	GC	NR	NR	NR
Wang	2016	China	90	65 (mean)	RT-PCR	>median	GC	I-IV	60	OS, DFS
Wang	2017	China	120	60	RT-PCR	Fold change > 1.5	GC	I-IV	NR	NR
Wu	2014	China	141	60 (mean)	RT-PCR	≥median	GC	I-IV	70	OS
Zhu	2018	China	180	60 (mean)	RT-PCR	Score ≥ 2	GC	I-IV	NR	NR
Liu	2018	China	80	65.1 (mean)	RT-PCR	2.072	GC	I-IV	NR	NR
Li	2018	China	42	NR	RT-PCR	≥median	GC	NR	NR	NR
Zhu	2019	China	40	NR	RT-PCR	≥median	GC	NR	NR	NR
Peng	2018	China	333	59.42 (mean)	RT-PCR	≥median	GC	I-IV	60	OS, PFS
Xu	2014	China	105	55 (mean)	RT-PCR	T/N ≥ 2	EC	I-IV	34.5 (median)	OS, PFS
Bai	2017	China	89	58 (mean)	RT-PCR	≥0.2909	EC	I-IV	NR	NR
Tan	2015	China	58	NR	RT-PCR	≥median	PC	NR	NR	OS
Qu	2014	China	39	65 (mean)	RT-PCR	NR	PC	I-IV	NR	NR
Zou	2019	China	129	60 (mean)	RT-PCR	>median	PC	I-IV	NR	OS
Hu	2016	China	63	NR	RT-PCR	≥median	PC	NR	NR	NR

Abbreviations: NR: not reported; RT-PCR: real-time polymerase chain reaction; T/N: tumor/normal; CRC: colorectal cancer; EC: esophagus cancer; GC: gastric cancer; PC: pancreatic cancer; LC: liver cancer; OS: overall survival; DFS: disease-free survival; RFS: recurrence-free survival; PFS: progression-free survival.

**Table 2:** Newcastle-Ottawa Scale for each included study.

Study	Selection	Comparability	Exposure	Total quality score
Yamada, 2015	3	1	3	7
Kahlert, 2011	3	2	3	8
Cellura, 2015	3	0	3	6
Huang, 2015	3	2	3	8
Jiang, 2017	3	2	3	8
Mastumura, 2015	4	2	3	9
Cruz-Gil, 2018	3	1	3	7
Koga, 2010	3	1	3	7
Zhu, 2017	3	2	3	8
Zhang, 2018	3	3	3	9
Yin, 2019	3	3	2	8
Marcuello, 2019	3	3	3	9
Guo, 2014	3	2	2	7
Han, 2012	3	2	3	8
Hu, 2018	3	0	3	6
Hung, 2015	3	2	3	8
Yu, 2016	3	1	3	7
Zhang, 2015	3	2	3	8
Zhu, 2010	3	2	2	7
Jiang, 2018	3	2	2	7
Cai, 2016	3	2	3	8
Li, 2014	4	2	3	9
Ibarrola-Villava, 2015	3	2	3	8
Wang, 2016	4	2	3	9
Wang, 2017	4	2	3	9
Wu, 2014	3	2	3	8
Zhu, 2018	3	2	3	8
Liu, 2018	3	3	3	9
Li, 2018	3	2	2	7
Zhu, 2019	2	3	2	7
Peng, 2018	3	3	3	9
Xu, 2014	4	2	3	9
Bai, 2017	3	2	3	8
Tan, 2015	3	0	3	6
Qu, 2014	2	1	3	6
Zou, 2019	3	2	3	8
Hu, 2016	3	1	3	7

**Table 3:** Subgroup analyses were stratified on the basis of histology.

	Stage		Grade		Lymph node metastasis		Distant metastasis	
Colorectal cancer	*N*	OR (95% CI)	*N*	OR (95% CI)	*N*	OR (95% CI)	*N*	OR (95% CI)
	6	2.74 (1.45, 5.18)	3	1.36 (0.74, 2.51)	7	1.89 (0.99, 3.63)	8	2.02 (0.77, 5.32)
Gastric cancer	*N*	OR (95% CI)	*N*	OR (95% CI)	*N*	OR (95% CI)	*N*	OR (95% CI)
	2	0.42 (0.17, 1.04)	2	0.31 (0.14, 0.70)	3	0.46 (0.14, 1.52)	1	0.31 (0.10, 0.97)
Esophagus cancer	*N*	OR (95% CI)	*N*	OR (95% CI)	*N*	OR (95% CI)	*N*	OR (95% CI)
	1	1.72 (0.95, 3.12)	1	1.65 (0.86, 3.16)	1	1.87 (0.91, 3.85)	None	None
Liver cancer	*N*	OR (95% CI)	*N*	OR (95% CI)	*N*	OR (95% CI)	*N*	OR (95% CI)
	4	0.66 (0.18, 2.45)	4	0.80 (0.47, 1.35)	1	4.75 (1.37, 16.47)	None	None
Pancreatic cancer	*N*	OR (95% CI)	*N*	OR (95% CI)	*N*	OR (95% CI)	*N*	OR (95% CI)
	None	None	None	None	None	None	None	None

Abbreviations: *N*: study numbers.

## Supplementary Materials

Supplementary 1 Figure 1: funnel plots of publication bias in the meta-analysis as shown in Figure 2.

Supplementary 2 Figure 2: funnel plots of publication bias in the meta-analysis of miR-19 expression and tumor stage.

Supplementary 3 Figure 3: funnel plots of publication bias in the meta-analysis of miR-19 expression and tumor differentiation degree.

Supplementary 4 Figure 4: funnel plots of publication bias in the meta-analysis of miR-19 expression and lymph node metastasis.

Supplementary 5 Figure 5: funnel plots of publication bias in the meta-analysis of miR-19 expression and distant metastasis.

Supplementary 6 Figure 6: funnel plots of publication bias in the meta-analysis of miR-19 expression and OS as shown in Figure 8.

Supplementary 7 Figure 7: funnel plots of publication bias in the meta-analysis of miR-19 expression and DFS as shown in Figure 10.
